# Trends in Clinically Significant Pain Prevalence Among Hospitalized Cancer Patients at an Academic Hospital in Taiwan: A Retrospective Cohort Study

**DOI:** 10.1097/MD.0000000000002099

**Published:** 2016-01-08

**Authors:** Wei-Yun Wang, Shung-Tai Ho, Shang-Liang Wu, Chi-Ming Chu, Chun-Sung Sung, Kwua-Yun Wang, Chun-Yu Liang

**Affiliations:** From the Graduate Institute of Medical Sciences (W-YW, K-YW, C-YL), National Defense Medical Center; Department of Nursing (W-YW), Tri-Service General Hospital; Department of Anesthesiology (S-TH, C-SS), Taipei Veterans General Hospital; Taiwan Research Association of Health Care (S-LW); School of Public Health (C-MC), National Defense Medical Center; School of Medicine (C-SS), National Yang-Ming University; Department of Nursing (K-YW), Taipei Veterans General Hospital; and School of Nursing (K-YW, C-YL), National Defense Medical Center, Taipei, Taiwan.

## Abstract

Clinically significant pain (CSP) is one of the most common complaints among cancer patients during repeated hospitalizations, and the prevalence ranges from 24% to 86%. This study aimed to characterize the trends in CSP among cancer patients and examine the differences in the prevalence of CSP across repeated hospitalizations.

A hospital-based, retrospective cohort study was conducted at an academic hospital. Patient-reported pain intensity was assessed and recorded in a nursing information system. We examined the differences in the prevalence of worst pain intensity (WPI) and last evaluated pain intensity (LPI) of ≥4 or ≥7 points among cancer inpatients from the 1st to the 18th hospitalization. Linear mixed models were used to determine the significant difference in the WPI and LPI (≥4 or ≥7 points) at each hospitalization.

We examined 88,133 pain scores from the 1st to the 18th hospitalization among cancer patients. The prevalence of the 4 CSP types showed a trend toward a reduction from the 1st to the 18th hospitalization. There was a robust reduction in the CSP prevalence from the 1st to the 5th hospitalization, except in the case of LPI ≥ 7 points. The prevalence of a WPI ≥ 4 points was significantly higher (0.240-fold increase) during the 1st hospitalization than during the 5th hospitalization. For the 2nd, 3rd, and 4th hospitalizations, there was a significantly higher prevalence of a WPI ≥ 4 points compared with the 5th hospitalization. We also observed significant reductions in the prevalence of a WPI ≥ 7 points during the 1st to the 4th hospitalizations, an LPI ≥ 4 points during the 1st to the 3rd hospitalizations, and an LPI ≥ 7 points during the 1st to the 2nd hospitalization.

Although the prevalence of the 4 CSP types decreased gradually, it is impossible to state the causative factors on the basis of this observational and descriptive study. The next step will examine the factors that determine the CSP prevalence among cancer patients. However, based on these positive findings, we can provide feedback to nurses, physicians, and pharmacists to empower them to be more committed to pain management.

## INTRODUCTION

Pain is one of the most feared and burdensome symptoms experienced during repeated hospitalizations among cancer patients.^1,2^ Cancer patients repeatedly require hospitalization to receive professional care, and cancer-related care services are usually performed in 3 stages. Patients undergo surgery, chemotherapy, and/or radiation therapy after the cancer diagnosis (the initial stage), and continue receiving chemotherapy and other treatments after this initial stage (the continuing stage). Eventually, palliative and/or hospice care are provided to cancer patients to minimize their pain before death (the final stage).^3^ However, the prevalence of pain among cancer patients ranges from 24% to 86%,^1,4^ and more than one-third of patients with pain grade their pain as moderate or severe. Despite clear World Health Organization (WHO) recommendations, pain still is a major problem experienced by cancer patients.^1^

When attempting to calculate pain prevalence in the hospital setting, simply asking a patient whether they are experiencing pain is not adequate because the severity of pain is not determined.^1^ Patient-reported pain intensity can be assessed using a 0 to 10 numerical rating scale (NRS); the Faces Pain Scale (FPS); the Faces, Legs, Activity, Cry, and Consolability (FLACC) Behavioral Tool; or a visual analogue scale (VAS), which are important tools that quantify a patient's perception of pain.^4–9^ “Clinically significant pain” (CSP) is defined as patient-reported pain intensity >4 points.^10^ In particular, pain intensity ≥7 points is defined as severe pain.^4,11,12^ To clearly document patient-reported pain intensity and to make the data available in real time, an electronic nursing information system (NIS) must be established to record and collect pain intensity, rather than relying on traditional manual chart documentation.

Although previous studies have used pain prevalence as an important indicator of pain in patients,^4,13^ no data are currently available regarding CSP prevalence, which can be examined among cancer patients according to the worst pain intensity (WPI) and the last evaluated pain intensity (LPI) before discharge for each hospitalization. Moreover, the literature on pain assessment and management among hospitalized cancer patients remains limited.^2^ In addition, traditional manual chart reviews and interviews are still the most common methods for data collection.^4,13^ Therefore, if we want to conduct time-series-based and hospital-based outcome analyses among patients, an electronic NIS is superior to the traditional methods of data collection. In terms of the cutoff points of 4 and 7, we divided CSP into 4 types: moderate-to-severe pain with a WPI ≥ 4 points; moderate-to-severe pain with an LPI ≥ 4 points; severe pain with a WPI ≥ 7 points; and severe pain with an LPI ≥ 7 points. Thus, the purposes of this study were to characterize trends in the prevalence of the 4 types of CSP among cancer patients during each hospitalization and to examine differences in the CSP prevalence across repeated hospitalizations based on an electronic NIS.

## MATERIALS AND METHODS

### Design

This study was a single-center, hospital-based, retrospective cohort study conducted at a national academic hospital in Taiwan. All cancer inpatients admitted between January 1, 2011 and December 31, 2013 were included in this analysis. According to the International Classification of Diseases, 9th Revision, the codes 140–209 and 230–239 were used to identify cancer patients.

For each patient during each hospitalization, we selected only one pain score, which included 2 types: WPI and LPI before discharge. Patient-reported pain intensity scores >4 points were defined as CSP. In particular, a pain intensity ≥ 7 points was defined as severe pain.^4,11,12^ Considering the cutoff points of 4 and 7, the prevalence of CSP or severe pain among the cancer patients was examined via the WPI and LPI during each hospitalization. CSP during each hospitalization included 4 types: moderate-to-severe pain with a WPI ≥ 4 points; moderate-to-severe pain with an LPI ≥ 4 points; severe pain with a WPI ≥ 7 points; and severe pain with an LPI ≥ 7 points. The formula for calculating the CSP prevalence was as follows: CSP prevalence at each hospitalization = number of patients with WPI (LPI) scores ≥ 4 (moderate-to-severe pain) or ≥ 7 points (severe pain)/total number of inpatients for this hospitalization.

### Setting

The hospital has 2747 inpatient beds and approximately 2700 nursing staff members who provide health care services. This hospital manages approximately 97,000 hospitalizations per year. An electronic NIS is used for the routine documentation and charting of vital signs, including pain intensity scores; information is recorded at bedside using hand-held devices. During each observation, the nurses immediately (at the patient's bedside) recorded the patient-reported pain intensity in the NIS. This immediate data entry ensured the accuracy of the recorded pain intensities. This NIS was introduced at the academic hospital in 2011 and has been used hospital-wide for all inpatients since then. Before 2011, all nurses participated in an educational program on proper pain assessment and pain documentation via an NIS. The training program was the cornerstone of quality pain assessment and pain documentation.

Chen^14^ analyzed the integrity of the NIS records of cancer inpatient pain intensities from January 1 to December 31, 2012. The study used 4 levels of analysis (the number of assessments per person, the number of days of hospitalization per person, the number of hospitalizations per person, and the number of people assessed). The analysis revealed high integrity (97.3%–99.9%) for the database. Therefore, the integrity of the pain assessment database could be ensured by integrating pain assessment sources.

### Data Collection

Patient-reported pain intensity was evaluated with an NRS and the FPS in alert and cooperative patients and with the FLACC Behavioral Tool in unconscious or uncooperative patients.^6–8^ These 3 pain scales, which have good reliability and validity, are all 11-point pain scales where 0 points indicates no pain at all and 10 points indicates the worst possible pain.^7,8^ All patients were assessed systematically at least once per day by the nurses. Specifically, the pain assessment was performed once each day when no pain at all was present or when the pain intensity was <4 and tolerable, thrice per day when the pain intensity was ≥4 or <4 and intolerable, and as needed when painkiller therapy implementation was indicated.

### Ethical Considerations

This study was conducted with the approval of the Institutional Review Board of Taipei Veterans General Hospital. The research was supported by the chief executive officer, medical director and nursing officer of the study hospital.

### Analytic Approach

Descriptive statistics were generated for CSP prevalence from the 1st to the 18th hospitalization, and the prevalence of the 4 CSP types is shown in Table 1. From the 1st to 5th hospitalizations, the prevalence of the 4 CSP types sharply decreased, and the absolute differences compared with the previous prevalence was greater than or equal to the average absolute prevalence differences during the 1st to 18th hospitalizations. Therefore, we selected the 5th hospitalization as the reference point for determining the differences in the prevalence of WPI and LPI (≥ 4 or ≥ 7 points) at each hospitalization using linear mixed models for inferential statistics. A *P* value <0.05 was considered significant. Statistical analyses were performed with the Statistical Package for the Social Sciences (SPSS) version 21.0 for Windows (SPSS Inc, Chicago, IL).

**TABLE 1:**
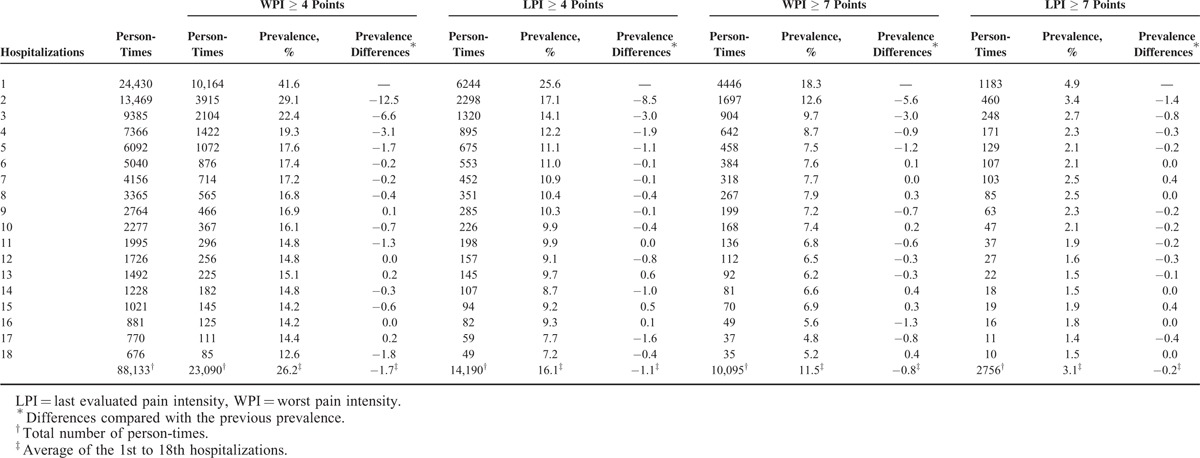
The Prevalence of Clinically Significant Pain Among Cancer Inpatients During Each Hospitalization

## RESULTS

**FIGURE 1:**
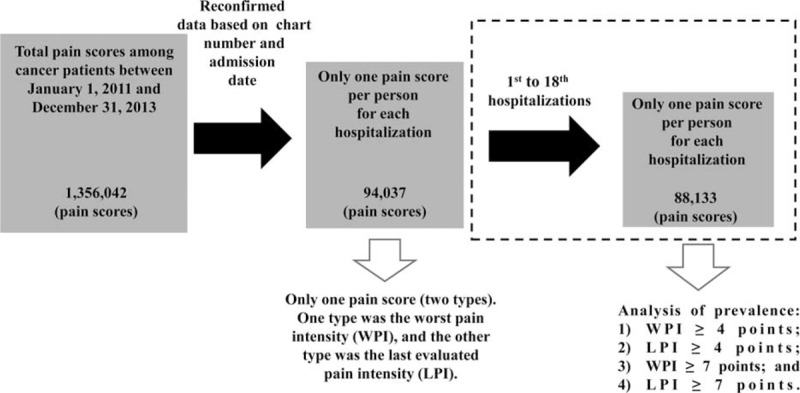
Retrieval of clinically significant pain scores from the NIS database. NIS = nursing information system.

A total of 1,356,042 pain scores were collected over the 3-year time period. After we reconfirmed the data based on the chart number and admission date, the number of pain scores was reduced to 94,037. We determined the CSP prevalence during each hospitalization, and the number of hospitalizations per patient at this hospital ranged from 1 to 18. In total, 88,133 pain scores were studied. The process of retrieving CSP information from the NIS database is shown in Figure 1.

### Demographic Characteristics of the Inpatients With Cancer

We examined 88,133 pain scores from the 1st to the 18th hospitalization of patients with cancer. The inpatient age ranged from 0.3 to 101.6 years with a mean of 59.3 ± 17.1 years. Among the inpatients, 47,773 (54.2%) were males and 40,360 (45.8%) were females.

### Trends in CSP Prevalence

As the number of hospitalizations increased, the prevalence of a WPI ≥ 4 points decreased from 41.6% to 12.6%, and the prevalence of a WPI ≥ 7 points decreased from 18.3% to 5.2%. Regarding LPI, we also observed downward trends in pain prevalence. With increasing hospitalizations, the prevalence of an LPI ≥ 4 points decreased from 25.6% to 7.2%, and the prevalence of an LPI ≥ 7 points decreased from 4.9% to 1.5%. There was a robust reduction in the CSP prevalence from the 1st to the 5th hospitalization, except for an LPI ≥ 7 points (Table 1).

### Differences in CSP Prevalence During Each Hospitalization

**FIGURE 2:**
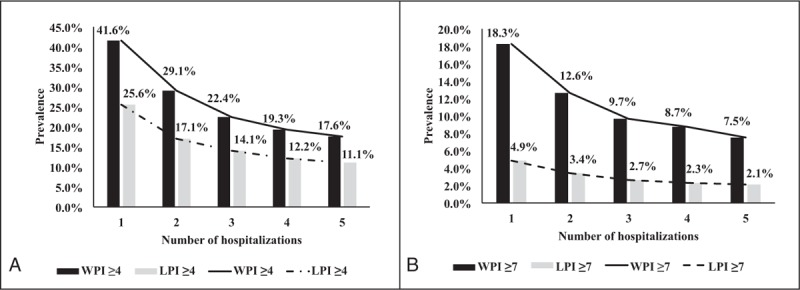
Trends in the CSP prevalence from the 1st to the 5th hospitalization. The 5th hospitalization was viewed as a reference group to determine the CSP differences for WPI and LPI of (A) ≥4 or (B) ≥7 points using linear mixed models. (A) For WPI ≥ 4, 1st versus 5th times (*P* < 0.001); 2nd versus 5th times (*P* < 0.001); 3rd versus 5th times (*P* < 0.001); and 4th versus 5th times (*P* = 0.021). For LPI ≥ 4, 1st versus 5th times (*P* < 0.001); 2nd versus 5th times (*P* < 0.001); 3rd versus 5th times (*P* < 0.001); and 4th versus 5th times (*P* = 0.088). (B) For WPI ≥ 7, 1st versus 5th times (*P* < 0.001); 2nd versus 5th times (*P* < 0.001); 3rd versus 5th times (*P* < 0.001); and 4th versus 5th times (*P* = 0.027). For LPI ≥ 7, 1st versus 5th times (*P* < 0.001); 2nd versus 5th times (*P* < 0.001); 3rd versus 5th times (*P* = 0.063); and 4th versus 5th times (*P* = 0.490). CSP = clinically significant pain, LPI = last evaluated pain intensity, WPI = worst pain intensity.

The prevalence of a WPI ≥ 4 points during the 1st hospitalization was significantly higher (0.240-fold increase) than the 5th hospitalization. For the 2nd, 3rd and 4th hospitalizations, there was a significantly higher prevalence of a WPI ≥ 4 points compared with the 5th hospitalization. After the 11th hospitalization, the prevalence of a WPI ≥ 4 points was significantly lower than the prevalence for the 5th hospitalization. In addition, the prevalence of an LPI ≥ 4 points was significantly higher during the 1st (0.145-fold increase), 2nd (0.060-fold increase), and 3rd (0.030-fold increase) hospitalizations (Table 2). Table 2 also shows the prevalence of a WPI or LPI ≥ 7 points. To evaluate the CSP prevalence from the 1st to the 5th hospitalization, we characterized the trends in the prevalence of the 4 CSP types (Fig. 2).

**TABLE 2:**
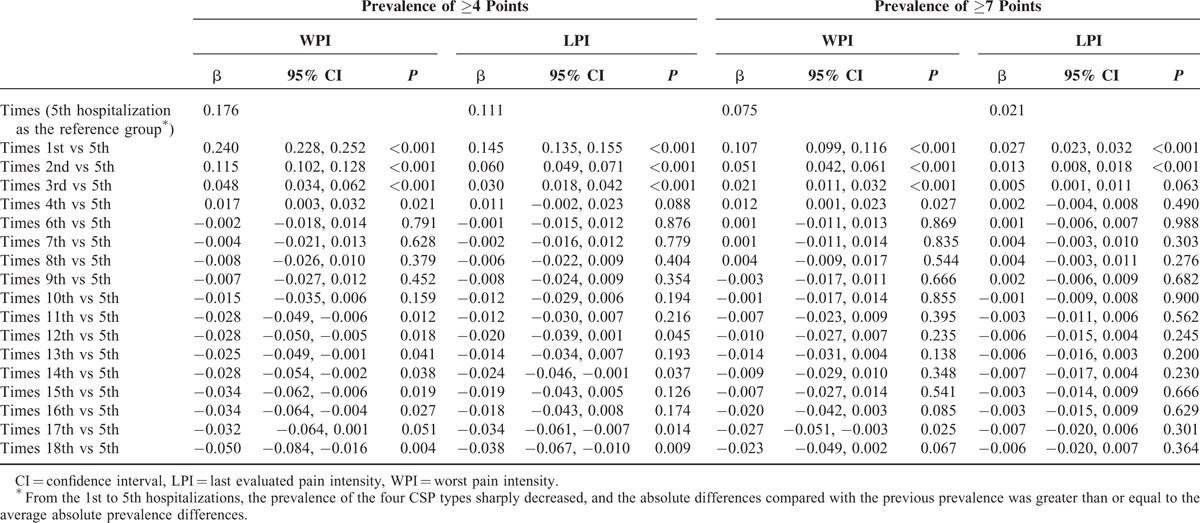
Differences in the Clinically Significant Pain Prevalence Among Cancer Inpatients During Each Hospitalization

## DISCUSSION

In this study, we examined the CSP prevalence during each hospitalization among cancer patients who were repeatedly hospitalized over time. Importantly, we found that the prevalence of the 4 CSP types decreased from the 1st to the 18th hospitalization. Specifically, the prevalence of a WPI ≥ 4 or 7 points was significantly reduced from the 1st to the 4th hospitalization. The prevalence of an LPI ≥ 4 points was significantly reduced from the 1st to the 3rd hospitalization, and the prevalence of an LPI ≥ 7 points was significantly reduced from the 1st to the 2nd hospitalization.

In the present study, the prevalence of a WPI ≥ 4 points ranged from 12.6% to 41.6%, and the prevalence of a WPI ≥ 7 points ranged from 5.2% to 18.3%. These values are similar to other studies ^4,7^ and lower than the values published in a systematic review of cases over the past 40 years.^1^ However, the previous studies focused on pain prevalence during a single hospitalization. Because cancer patients often require repeated hospitalizations, examining pain prevalence during one hospitalization is insufficient for these inpatients. Therefore, a long-term analysis of pain during the repetitive hospitalizations of each patient should be performed.

In addition, we assessed LPI before discharge and demonstrated that the prevalence of an LPI ≥ 4 or 7 points was lower than the prevalence of a WPI ≥ 4 or 7 points for each hospitalization. The CSP prevalence tended to decrease before discharge for each hospitalization. At our institution, after each pain assessment, the nurses used proper painkiller therapy to prevent the severity of the pain from worsening; however, to date, there is still a gap between pain assessment and the implementation of pain treatment strategies. To reduce the clinical divide, high-quality pain documentation is useful because the assessment and documentation of pain are viewed as the cornerstones of effective pain management.^10^ Standard-setting agencies, such as the Joint Commission, rely on documentation in the patient care record to assess the quality of pain management.^15,16^ However, over one-third of the information recorded is not in accord with the patient's report, and the nurses’ documentation regarding pain may be incomplete in the nursing records.^11^ Therefore, we used systematic pain assessments and regularly documented pain intensity by direct entry into an NIS database in our hospital. Nurses specifically recorded the pain score in the NIS, so that the pain intensity and effectiveness of the chosen pain management therapy could be rapidly determined by nurses, physicians, and pharmacists.^17^ Through systematic pain assessment and documentation in an NIS, nurses are more attentive to pain symptoms among cancer patients and can immediately notify the physician in charge to improve pain. At our institution, pain assessment and management are addressed through a multidisciplinary approach. Nurses, physicians, and pharmacists all facilitate pain management; importantly, the nurses are the gatekeepers in pain assessment and management.

All cancer patients will experience pain during repeated hospitalizations.^10^ Carr et al^10^ noted that the recurrence of moderate or severe pain during repeated hospitalizations reflects a lack of continuous and effective pain management strategies. In our hospital, the prevalence of a WPI ≥ 4 and 7 points was significantly reduced from the 1st to the 4th hospitalization. The prevalence of an LPI ≥ 4 points was significantly reduced from the 1st to the 3rd hospitalization, and the prevalence of an LPI ≥ 7 points was significantly reduced from the 1st to the 2nd hospitalization. This observation affirmed the significantly decreased CSP prevalence during repeated hospitalizations, which could be the long-term outcome of the nurses’ role as gatekeepers in pain assessment and management for cancer inpatients.

Our study had 2 methodological strengths. First, we used an NRS, the FPS, or the FLACC Behavioral Tool to measure an individual's pain intensity, which is also known as a patient-reported outcome. Patient-reported outcomes are important measurements that have been incorporated into ongoing clinical care.^18,19^ This implies that during daily practice, simply asking “the pain question” (without the use of extensive and time-consuming questionnaires) can detect patients who are experiencing pain. Based on the patient-reported pain intensity, we could characterize the trends in CSP prevalence during repeated hospitalizations. Using the same scoring method at different time points to measure pain allows clinicians to observe variation in pain over time.^5,19^ However, cancer pain is a complex and multidimensional symptom that is affected by psychological and social variables and the disease process itself.^20^ Although the distinction between the presence or absence of CSP among cancer patients will enable the calculation CSP prevalence, this distinction does not provide information about the severity of pain or the degree of pain reduction between the WPI and LPI for each hospitalization. To facilitate the comparison of studies and to coordinate the planning of pain services, multidimensional tools, such as the absolute difference in pain intensity or the percentage difference in pain intensity, may be used in future research.

The second strength of this study was the use of an electronic data capture system for outcome studies that integrated data collection into the ongoing process of patient care to conduct a hospital-based study. In general, there is still a lack of information about pain assessment and scoring on a hospital-wide basis.^10^ This gap presents a challenge, and it can be difficult to integrate the collection of valid outcome measures into a busy clinical practice in which time and cost-containment pressures already exist. The real-time availability of data essentially requires electronic data capture followed by automatic reporting. The burden of providing the data on either the patient or the physician must be minimized to make data collection as brief as possible to facilitate meaningful results. Therefore, the development and implementation of patient-reported outcome data collection systems for a large number of pain programs and integration into electronic health records are critical steps.^18^ Then, patient-reported pain intensity can be clearly documented, eliminating transcription error, facilitating the subsequent retrieval and analysis of data, and allowing tracking over time by clinicians to guide patient care.^10^

Our study evaluated the CSP prevalence in an entire population of cancer inpatients during each hospitalization in an academic hospital, thereby addressing the weaknesses of the previous study,^7^ which evaluated the pain intensity of first-time medical oncology unit inpatients. In addition, most studies related to pain prevalence have relied on interviews, manual documentation of pain assessment, and retrospective chart reviews.^21–24^ In our study, the CSP prevalence was similar to or lower than the prevalence reported in other studies. This result may be due to the electronic NIS instead of the traditional data collection method. The most common electronic NIS functionalities or components are records of patient pain intensity and clinical notes.^25^ The NIS enables the integration of pain intensity data collection into the ongoing process of pain measurement, and provides comprehensive information about pain assessment.^18^ The electronic NIS is increasingly viewed as an essential tool for quality assurance and improvement in a variety of care settings.^25^ Further research regarding pain documentation using electronic medical records is needed.

This study also had 2 limitations. One limitation is that this study was conducted in a cancer inpatient cohort that was heterogeneous regarding clinical stage. This study was conducted at a single academic hospital. Thus, the second limitation is that the generalizability of the findings may be limited. However, our study design can be replicated at other institutions to validate these results.

## CONCLUSION

In conclusion, this report represents the first hospital-based study that used an electronic database to analyze CSP prevalence among cancer inpatients in Taiwan. The trend curves for the prevalence of the 4 CSP types indicated a reduction from the 1st to the 18th hospitalization. In particular, the prevalence of a WPI ≥ 4 and 7 points was significantly reduced from the 1st to the 4th hospitalization. Although the prevalence of the 4 CSP types decreased gradually, it is impossible to state the causative factors on the basis of this observational and descriptive study. The next step will examine the factors that determine the CSP prevalence among cancer patients. However, based on these positive findings, we can provide feedback to nurses, physicians, and pharmacists to empower them to be more committed to pain management.
